# Generation of Human-Like Movement from Symbolized Information

**DOI:** 10.3389/fnbot.2018.00043

**Published:** 2018-07-17

**Authors:** Shotaro Okajima, Maxime Tournier, Fady S. Alnajjar, Mitsuhiro Hayashibe, Yasuhisa Hasegawa, Shingo Shimoda

**Affiliations:** ^1^Department of Mechanical Science and Engineering, Graduate School of Engineering, Nagoya University, Nagoya, Japan; ^2^Intelligent Behavior Control Unit (BTCC), Brain Science Institute (BSI), RIKEN, Nagoya, Japan; ^3^College of IT, United Arab Emirates University, Al-Ain, United Arab Emirates; ^4^Department of Robotics, Tohoku University, Sendai, Japan

**Keywords:** mechanical resonance mode, tacit learning, control structure, symbolized information, human-like movement

## Abstract

An important function missing from current robotic systems is a human-like method for creating behavior from symbolized information. This function could be used to assess the extent to which robotic behavior is human-like because it distinguishes human motion from that of human-made machines created using currently available techniques. The purpose of this research is to clarify the mechanisms that generate automatic motor commands to achieve symbolized behavior. We design a controller with a learning method called tacit learning, which considers system–environment interactions, and a transfer method called mechanical resonance mode, which transfers the control signals into a mechanical resonance mode space (MRM-space). We conduct simulations and experiments that involve standing balance control against disturbances with a two-degree-of-freedom inverted pendulum and bipedal walking control with humanoid robots. In the simulations and experiments on standing balance control, the pendulum can become upright after a disturbance by adjusting a few signals in MRM-space with tacit learning. In the simulations and experiments on bipedal walking control, the robots realize a wide variety of walking by manually adjusting a few signals in MRM-space. The results show that transferring the signals to an appropriate control space is the key process for reducing the complexity of the signals from the environment and achieving diverse behavior.

## 1. Introduction

Can robots be good neighbors? Despite much effort by many researchers to make robots be good partners, robotic systems remain limited to being merely useful tools in factories and houses^1^. This is the case even though mobility control for rough terrain^2^and artificial intelligence for understanding human speech^3^,^4^ and behavior have improved drastically in recent years. What is the critical difference that distinguishes people from human-made machines?

We think that an important function that is currently missing from robotic systems is a way to create behavior from symbolized information. For instance, when walking, we deliberately attend to symbolized behavioral purposes such as “walk faster” and “turn right” or more symbolic forms such as “go to the station.” The detailed control signals that create such motion, such as joint trajectories and muscle activations, are then generated automatically.

It is said that these automatic control signals are created by the activities of local neural systems including the cerebellum and spinal cord. In our daily lives, we attend only to symbolized behavioral purposes that are highly specialized. The appropriate behavior and detailed control signals that achieve those purposes are then chosen according to the prevailing situation and the surrounding environment. If we could share such symbolized behavioral purposes with robots, and if the robots and we could create the appropriate behavior independently according to not only the surroundings but also the features of our respective functions, then we would feel that the robots are really our partners. Therefore, generating behavior from symbolized purpose could be an important way to assess the extent to which robots could be our partners with human-like behavior.

In this paper, we discuss the process of creating behavior from symbolized behavioral purpose, focusing on creating motor commands from simple behavioral targets through body–environment interactions. There have been various attempts to clarify the mechanisms that generate automatic motor commands from symbolized purposes, an important approach being a physiological one. Recently, Takei et al. (2017) reported the existence of neurons in the spinal cords of monkeys that commonly activate in association with various hand actions, suggesting that a small control signal, with dimensionality lower than the number of muscles, can encode complicated hand motion.

Model-based approaches provide the conceptual basis for the aforementioned physiological approach. A bow-tie structure (Csete and Doyle, 2004; Zhao et al., 2006) has been proposed to represent a biological control system whereby information acquired from the environment is gradually symbolized to reduce its dimensions, while control signals are created from this symbolized information to increase their dimension. The notions of muscle synergy (Bernstein, 1967; Tresch et al., 1999; d'Avella et al., 2003; Chvatal et al., 2011; Alnajjar et al., 2013; Barroso et al., 2014; Gonzalez-Vargas et al., 2015; Garcia et al., 2018; Kogami et al., 2018) and joint synergy (Schenkman et al., 1990; Latash, 2000; Yamasaki and Shimoda, 2016) that represent the output side of this bow-tie structure are prominent examples of estimating lower-dimensional signals from observable signals such as electromyographic signals. The sensor synergy representing the input side of the bow-tie structure has been discussed regarding estimating sensor signals from the environment (Ting, 2007; Latash, 2008; Alnajjar et al., 2015).

Another important approach to clarifying the mechanisms that generate automatic motor commands is the development of artificial controllers that have the same features as those of biological controllers. The autoencoders discussed in artificial intelligence (Hinton and Salakhutdinov, 2006; Hosseini-Asl et al., 2016) share the same idea as the bow-tie structure. Recently, there have been various discussions about using autoencoders to control robots (Noda et al., 2014; Finn et al., 2016; van Hoof et al., 2016; Kondo and Takahashi, 2017). KullbackLeibler control (Todorov, 2009) is an interesting task-dependent approach to control robot (Uchibe and Doya, 2014; Matsubara et al., 2015) with combination of control policies.

These computational approaches clarified that small control signals, with dimensionality lower than the number of motors, can represent behavioral features, suggesting that lower-dimensional control signals play the role of symbolized behavioral purposes. Shimoda et al. proposed a bio-mimetic behavior-adaptation architecture known as tacit learning (Shimoda and Kimura, 2010; Shimoda et al., 2013; Hayashibe and Shimoda, 2014) and have used it to generate bipedal walking from a roughly defined walking gait (Shimoda et al., 2013), to control the wrist joint of a forearm prosthesis in response to the wearer's shoulder movements (Oyama et al., 2016), and to control a lower-limb exoskeleton robot in response to the wearer's movements (Shimoda et al., 2015). Through experiments on this tacit learning adaptation, they established that two types of adaptation process could work simultaneously to adapt the behavior to an unorganized environment. One of these processes is selecting appropriate behavior and the other is adapting reactive behavior to unpredictable disturbances and small changes in body parameters and environment without changing the behavioral purpose.

Even though it has been established that it is important for these two processes to operate in parallel, the conditions of the controllers needed to realize such adaptation remain under discussion. Herein, we advance this discussion by using an artificial controller that can adapt the motor commands to real-time changes in the symbolized purpose, and we clarify the conditions for adapting in real time to both the environment and the symbolized purpose. We begin in section 2 by designing a controller with tacit learning and that transfers the control signals into a different control space know as mechanical resonance mode space (MRM-space). In MRM-space, the signals are used to control the mechanical resonance modes of the robot. This makes it easy to understand how the robot behavior changes when the control signal is changed in MRM-space. In sections 3 and 4, we propose an adaptation method in MRM-space using a two-degree-of-freedom (2DoF) inverted pendulum, 27DoF humanoid robot, and the NAO humanoid robot^5^, respectively. We show through simulation and experiment that this controller can adapt the motion to the environment. In section 5, we discuss the importance of body mechanisms in the process of simultaneous adaptation and how that process can be used to evaluate the human-like motion of a robot.

## 2. Methods used to design a control structure

Transfer to MRM-space and behavior adaptation by tacit learning are the key analytical methods of the present study. Because both methods are discussed in detail elsewhere (mechanical resonance mode Kry et al., 2009; tacit learning Shimoda and Kimura, 2010; Shimoda et al., 2013; Hayashibe and Shimoda, 2014), we explain their essential points only briefly herein.

### 2.1. Mechanical resonance mode

**Figure 1:**
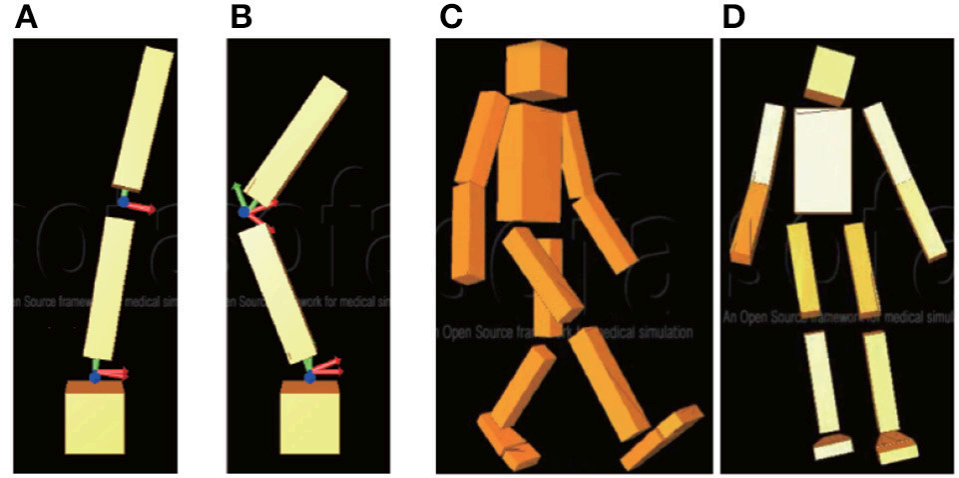
Modes of two-degree-of-freedom (2DoF) inverted pendulum and 27DoF humanoid robot: **(A)** first mode of pendulum (same-phase posture); **(B)** second mode of pendulum (anti-phase posture); **(C)** first mode of robot (bipedal leg swinging); **(D)** eighth mode (leg swinging on frontal plane).

A mechanical resonance mode is defined by the position and the condition of the robot joints. For instance, a 2DoF inverted pendulum has two mechanical resonance modes as shown in Figures 1A,B. A mechanical resonance mode is characterized mathematically by mode vectors and eigenvalues. Writing the equation of motion of a 2DoF inverted pendulum as

(1) $$\begin{array}{lll} M\ddot{\theta }+\text{K}\theta & = & 0 \end{array}$$

(2) $$\begin{array}{lll} \Leftrightarrow \;\ddot{\theta } & = & -M^{-1}K\theta , \end{array}$$

where **θ** ∈ **R**^2^ implies the angles of the joints, the mode vectors and eigenvalues are calculated by a singular-value decomposition (SVD) of **M**^−1^**K**:

(3) $$\begin{array}{l} M^{-1}K\Rightarrow \{\begin{array}{ll} v_{1},v_{2} & \left(mode\;vectors\right)\\ \lambda_{1},\lambda_{2} & \left(eigenvalues\right) \end{array},\\ \;SVD \end{array}$$

where **M** ∈ **R**^2 × 2^ is the inertia matrix of the pendulum linearized around **θ** = **0** and **K** ∈ **R**^2 × 2^ is a stiffness matrix that has the spring coefficients of the joints on its main diagonal. The first mode (**v**_1_) corresponds to the smallest eigenvalue (λ_1_) and the second mode (**v**_2_) corresponds to the next-largest eigenvalue (λ_2_). The mode vector represents the shape of the pendulum oscillation.

The state variable **θ** of the pendulum can be represented as a superposition of the mode vectors as follows:

(4) $$\begin{array}{l} \theta =v_{1}w_{1}+v_{2}w_{2}=\left[\begin{array}{cc} v_{1} & v_{2} \end{array}\right]{\left[\begin{array}{cc} w_{1} & w_{2} \end{array}\right]}^{T}\\ ∴\theta =Tw\;\left(\Leftrightarrow \;w=T^{-1}\theta \right), \end{array}$$

where *w*_1_, *w*_2_ represent the weights of each mode vector and **T** can be defined as a transfer matrix. We can define the weights of the mode vectors as symbolized state variables in MRM-space.

The adjustment of the symbolized state variables is reflected in the movement of individual joints by the transfer matrix. This is much like the top-down process in people, namely changing one's behavior by means of symbolized information without having to attend to the actions of individual joints.

### 2.2. Tacit learning

Tacit learning is an adaptive learning method inspired by two features of living beings. First, living beings can perform adaptive behavior globally even though control is realized by only a summation of local nerve-cell firings. Second, adaptive learning and behavioral control are calculated in parallel; this is unlike machine learning, whose calculation is divided into a learning phase and an action phase.

To apply these features to artificial controller, action targets and the concept of “reflex” are used in tacit learning. The reflex plays a role in directing the movement of the controlled system toward a state in situations in which the system does not receive many environmental stimuli from a global perspective. By enhancing the reflex by accumulating reflex commands, the system can acquire a state autonomously through system–environment interactions, where there are fewer environmental stimuli without having to distinguish between the learning phase and the action phase.

Other learning methods use behavioral functions or teaching signals to adjust the controlled system behavior and achieve adaptive behavior in a top-down manner. In that sense, tacit learning can be defined as a bottom-up learning process, adjusting the behavior through system–environment interactions. However, it can control the system to achieve adaptive behavior from a global perspective. Herein, we use tacit learning to develop a bio-mimetic adaptation process.

## 3. Standing balance control with 2DoF inverted pendulum

In this section, we introduce the controller with tacit learning in MRM-space and apply it to standing balance control of a 2DoF inverted pendulum. We show that the tacit learning controller can maintain balance against larger disturbances than the case without learning.

### 3.1. Model of 2DoF inverted pendulum

**Figure 2:**
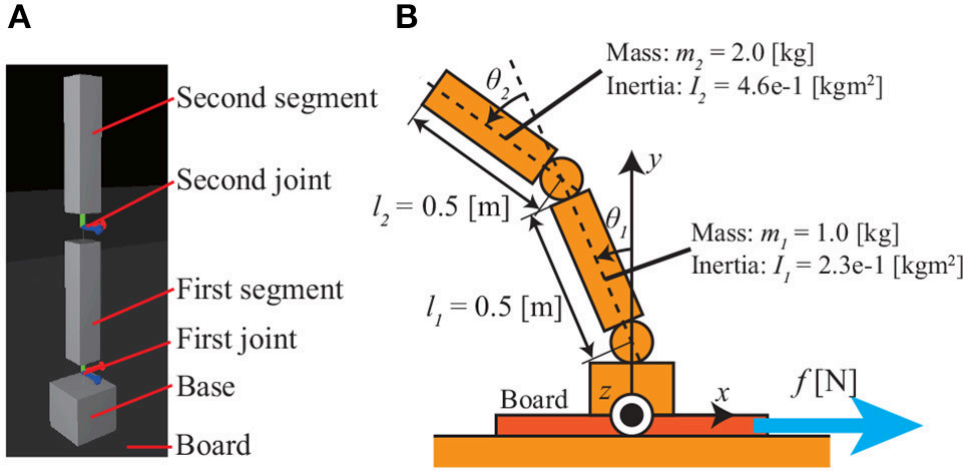
Model of 2DoF inverted pendulum: **(A)** pendulum model in simulator; **(B)** definitions of joint angles and system parameters. The pendulum sits on a board that is moved horizontally by a force *f* [N], thereby imparting disturbances to the pendulum.

Figure 2 shows the 2DoF inverted pendulum model used in this simulation. Its equation of motion is

(5) $$M(\theta )\ddot{\theta }+g(\theta ,\dot{\theta })=\tau ,$$

where **θ** is a 2 × 1 vector consisting of the joint angles, **τ** is a 2 × 1 torque vector that affects each joint, and **M**(**θ**) is a 2 × 2 inertia matrix given by

(6) $$\begin{array}{l} M\left(\theta \right)\\ =\left[\begin{array}{cc} I_{1}+m_{1}a_{1}^{2}+l_{1}^{2}m_{2}+\eta +2\xi \cos\theta_{2} & \eta +\xi \cos\theta_{2}\\ \eta +\xi \cos\theta_{2} & \eta \end{array}\right], \end{array}$$

where *a*_1_ = *l*_1_/2, *a*_2_ = *l*_2_/2, $\eta =I_{2}+m_{2}a_{2}^{2}$, *ξ* = *l*_1_*m*_2_*a*_2_, and $\text{g}\left(\theta ,\overset{\cdot }{\theta }\right)$ is

(7) $$\begin{array}{l} \begin{array}{c} g\left(\theta ,\overset{\cdot }{\theta }\right)\\ =\left[\begin{array}{c} -\xi \left(2{\overset{\cdot }{\theta }}_{1}+{\overset{\cdot }{\theta }}_{2}\right){\overset{\cdot }{\theta }}_{2}\sin\theta_{2}-g_{1}\sin\theta_{1}-g_{2}\sin\left(\theta_{1}+\theta_{2}\right)\\ \xi {\overset{\cdot }{\theta }}_{1}^{2}\sin\theta_{2}-g_{2}\sin\left(\theta_{1}+\theta_{2}\right) \end{array}\right], \end{array} \end{array}$$

where *g*_1_ = (*m*_1_*a*_1_ + *m*_2_*l*_1_)*g*, *g*_2_ = *m*_2_*a*_2_*g*, and *g* = 9.81 m/s^2^.

### 3.2. Standing balance control structure

**Figure 3:**
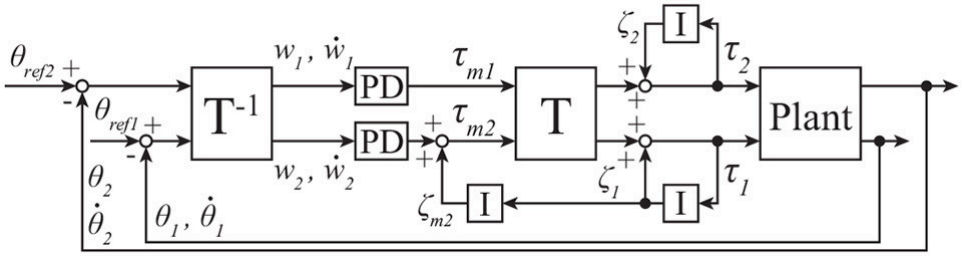
Block diagram for standing balance control of 2DoF inverted pendulum; see section 3.2 for details.

Figure 3 shows a standing balance controller designed by using the transfer matrix **T** described in 4 and tacit learning. Terms θ_*i*_ and τ_*i*_ are the angle and torque, respectively, of joint *i*. The torque vector **τ** for each joint is

(8) $$\begin{array}{l} \tau =TAT^{-1}\Delta \theta +TBT^{-1}\Delta \overset{\cdot }{\theta }+T\zeta_{m}+\zeta , \end{array}$$

(9) $$\begin{array}{l} \tau ={\left[\begin{array}{cc} \tau_{1} & \tau_{2} \end{array}\right]}^{T}. \end{array}$$

Δ**θ** and $\Delta \overset{\cdot }{\theta }$ are

(10) $$\begin{array}{l} \Delta \theta =\left[\begin{array}{c} \theta_{ref}-\theta \end{array}\right],\Delta \overset{\cdot }{\theta }=-\overset{\cdot }{\theta }, \end{array}$$

where **θ** and $\overset{\cdot }{\theta }$ are state variables:

(11) $$\begin{array}{l} \theta ={\left[\begin{array}{cc} \theta_{1} & \theta_{2} \end{array}\right]}^{T},\;\overset{\cdot }{\theta }={\left[\begin{array}{cc} {\overset{\cdot }{\theta }}_{1} & {\overset{\cdot }{\theta }}_{2} \end{array}\right]}^{T}. \end{array}$$

**θ**_*ref*_ is a reference for each joint:

(12) $$\begin{array}{l} \theta_{ref}={\left[\begin{array}{cc} \theta_{ref1} & \theta_{ref2} \end{array}\right]}^{T}. \end{array}$$

Terms **A** and **B** are diagonal matrices:

(13) $$\begin{array}{l} A=\left[\begin{array}{cc} k_{p1} & 0\\ 0 & k_{p2} \end{array}\right],\;B=\left[\begin{array}{cc} k_{d1} & 0\\ 0 & k_{d2} \end{array}\right], \end{array}$$

where *k*_*p*1_ and *k*_*p*2_ are proportional (P) gains of the proportional-derivative (PD) controller in MRM-space, *k*_*d*1_ and *k*_*d*2_ are derivative gains of the PD controller in MRM-space, and **T**^−1^ is the transfer matrix from joint space to MRM-space.

Term **ζ** is a vector that consists of the integration of **τ** as follows:

(14) $$\begin{array}{l} \zeta =\left[\begin{array}{c} \zeta_{1}\\ \zeta_{2} \end{array}\right]=\left[\begin{array}{cc} k_{1} & 0\\ 0 & k_{2} \end{array}\right]\left[\begin{array}{c} \int \tau_{1}dt\\ \int \tau_{2}dt \end{array}\right], \end{array}$$

where *k*_1_ and *k*_2_ are the coefficients of the integrators that accumulate the joint torques and output the integrated values. These accumulations correspond to tacit learning, and these integrators adjust the individual joint torques and work to keep the pendulum upright after disturbance through pendulum–environment interaction, as in Shimoda et al. (2013).

Term **ζ**_*m*_ a vector that consists of the integration of **ζ** as follows:

(15) $$\begin{array}{l} \zeta_{m}=\left[\begin{array}{c} \zeta_{m1}\\ \zeta_{m2} \end{array}\right]=\left[\begin{array}{cc} 0 & 0\\ k_{m2} & 0 \end{array}\right]\left[\begin{array}{c} \int \zeta_{1}dt\\ 0 \end{array}\right], \end{array}$$

where *k*_*m*2_ is the coefficient of the integrator in MRM-space that accumulates ζ_1_ and outputs the integrated values. *k*_*m*2_ can change the level of learning in standing balance control. *k*_*m*2_ = 0 is defined as “without learning,” and *k*_*m*2_ > 0 is defined as “with learning.” **ζ**_*m*_ adjusts the movement of the second mode, which we selected based on visual inspection of the movement of all modes. Because any disturbance has a pronounced effect on joint 1, the torque of the second mode is adjusted based on the torque of joint 1.

The whole system can be expressed by combining (5) and (8) as follows:

(16) $$\begin{array}{l} \begin{array}{c} M\left(\theta \right)\ddot{\theta }+g\left(\theta ,\overset{\cdot }{\theta }\right)=\;TAT^{-1}\Delta \theta +TBT^{-1}\Delta \overset{\cdot }{\theta }\\ \;+T\zeta_{m}+\zeta . \end{array} \end{array}$$

### 3.3. Standing balance control simulation and results

The two mode vectors **v**_1_, **v**_2_ of the pendulum defined in Figure 2 are given as

(17) $$\begin{array}{l} v_{1}=\left[\begin{array}{c} -0.9\\ -0.3 \end{array}\right],\;v_{2}=\left[\begin{array}{c} 0.3\\ -0.9 \end{array}\right], \end{array}$$

As shown in Figures 3A,B, the first mode **v**_1_ represents same-phase posture and the second mode **v**_2_ represents anti-phase posture. The transfer matrix **T** is

(18) $$\begin{array}{l} T=\left[\begin{array}{cc} -0.9 & 0.3\\ -0.3 & -0.9 \end{array}\right]. \end{array}$$

Standing balance control simulations are conducted as follows.

1. The pendulum is placed upright on a board that can move horizontally.
2. The board is moved for 0.2 s with the disturbance *f* [N].
3. A simulation is ended once the height of the center of mass(CoM) of the pendulum falls below 0.2 m or the pendulum become upright.

We conduct simulations with each of *f* = 170,… ,210 N. The gains are $k_{p1}=22.0,k_{d1}=21.0,k_{p2}=22.0,k_{d2}=21.0,k_{1}=1.0\times 10^{-3}$, and $k_{2}=1.0\times 10^{-3}$. The references are θ_*ref*1_ = θ_*ref*2_ = 0.0.

Table 1 gives the results of whether the pendulum falls down in the process of trying to maintain standing balance. The pendulum is clearly more stable with tacit learning in MRM-space than without tacit learning in MRM-space.

**Table 1:** Stability changes due to different coefficients.

**Disturbance**	**Without learning**	**With learning**
	***k*_*m*2_ = 0.0**	***k*_*m*2_ = 5.0 × 10^−4^**
170 ≤ *f* ≤ 184 [N]	Stable	Stable
185 ≤ *f* ≤ 204 [N]	Fallen	Stable
205 ≤ *f* [N]	Fallen	Fallen

**Figure 4:**
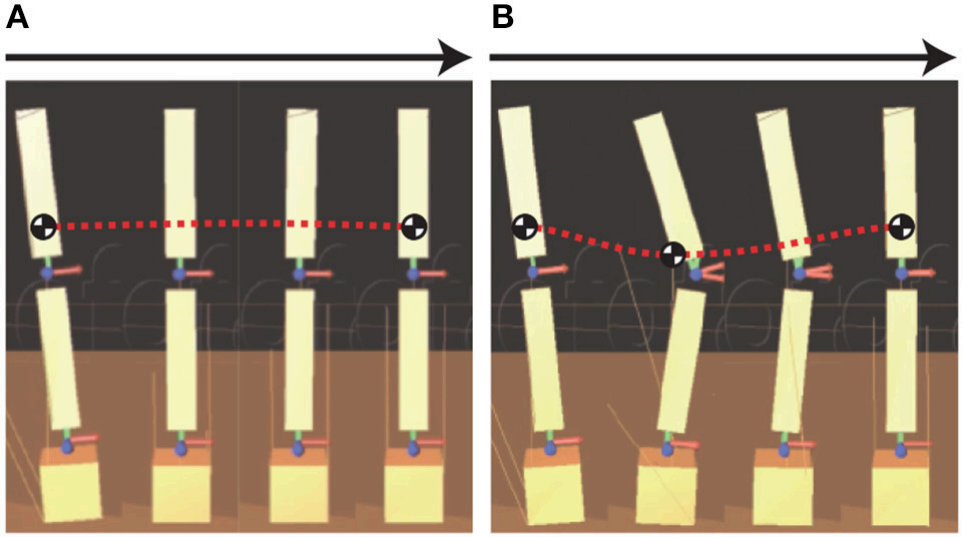
Overview of standing balance control simulation: **(A)** without learning; **(B)** with learning. “Without learning” means that tacit learning is applied to only joint space, and “With learning” means that tacit learning is applied to joint space and MRM-space. The red dotted line is the general trajectory of the center of mass (CoM) of the pendulum in the process of regaining balance after a disturbance. The CoM falls lower while regaining balance with tacit learning in MRM-space.

**Figure 5:**
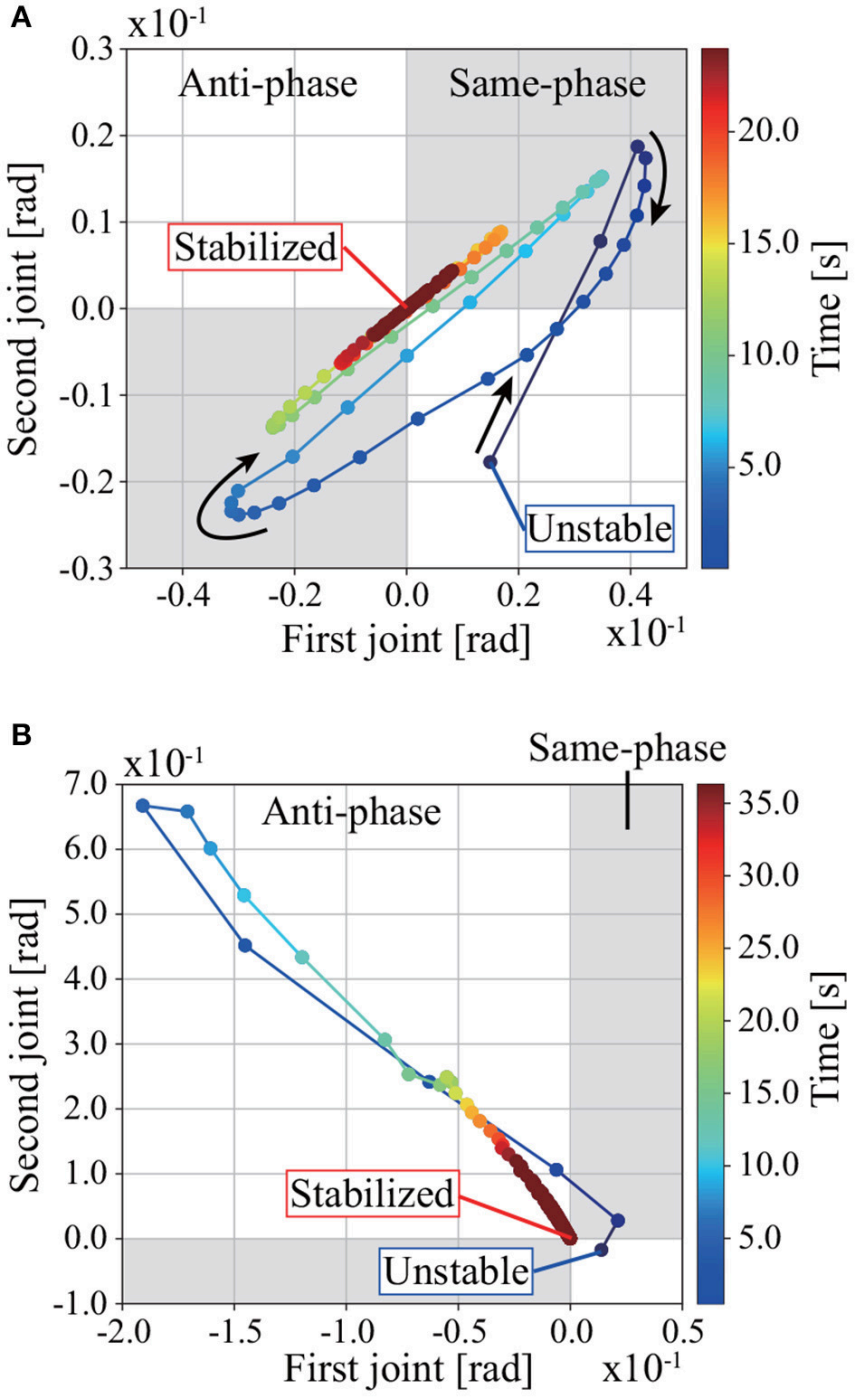
Trajectories of joints 1 and 2 while regaining balance in the simulation: **(A)** without learning; **(B)** with learning. The gray areas are where the joints move in phase, the white areas are where they move in anti-phase. Joints 1 and 2 move in phase while regaining balance without tacit learning in MRM-space. Joints 1 and 2 move in anti-phase while regaining balance with tacit learning in MRM-space. The disturbance is *f* = 184 N. The gain of tacit learning in MRM-space is *k*_*m*2_ = 5.0 × 10^−4^.

Figure 4 shows an overview of standing balance control simulations without and with learning in MRM-space. The pendulum CoM falls lower in the process of regaining balance when tacit learning is applied in MRM-space. Figure 5 shows the trajectories of joints 1 and 2 in the process of regaining balance. In the case without learning in MRM-space shown in Figure 5A, the pendulum becomes upright after the disturbance by moving joints 1 and 2 in phase. By contrast, in the case with learning in MRM-space shown in Figure 5B, joints 1 and 2 move in anti-phase.

**Figure 6:**
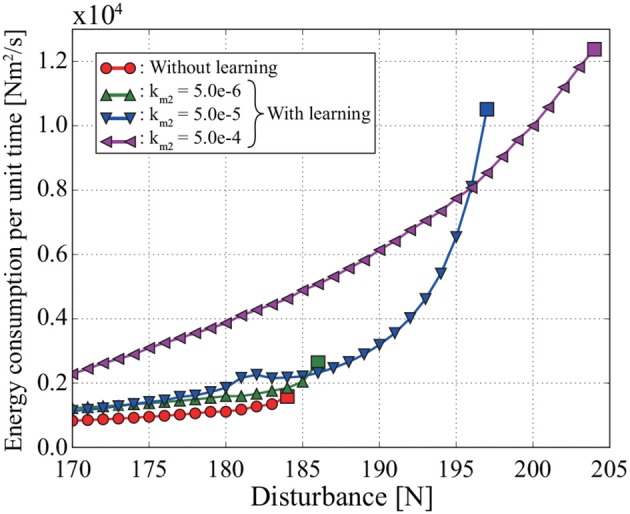
Relationship between energy consumption per unit time and disturbance in the simulation. Each square represents the maximum disturbance for which the pendulum can become upright. For example, the pendulum cannot become upright against a disturbance of 185 N or more without learning in MRM-space. Both the energy consumption per unit time and the stability against disturbance increase with the coefficient of tacit learning in MRM-space.

Figure 6 shows the relationship between the disturbance and the energy consumption *E* per unit time, which is calculated from

(19) $$\begin{array}{l} E=\frac{\sum_{t=0}^{T}\left({\tau_{1}}^{2}+{\tau_{2}}^{2}\right)}{T}, \end{array}$$

where *T* is the time until the pendulum becomes upright. We also calculate *E* when the simulation is conducted with tacit learning in MRM-space by using different integral coefficients, namely $k_{m2}=5.0\times 10^{-6}$ and 5.0 × 10^−5^.

The pendulum can become upright against a larger disturbance with learning in MRM-space than without learning in MRM-space. The size of disturbance that the pendulum can withstand without falling over increases with the integral coefficient *k*_*m*2_ for tacit learning. However, *E* also increases with *k*_*m*2_.

### 3.4. Standing balance control experiment and results

**Figure 7:**
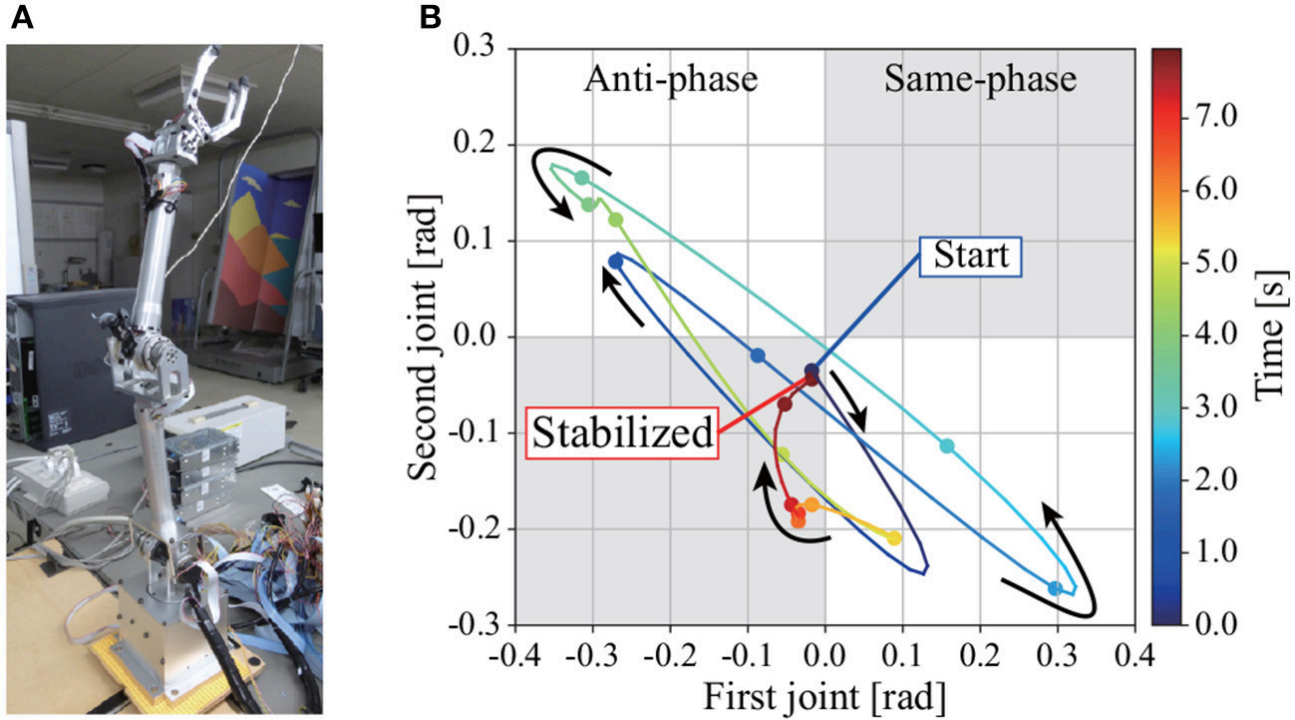
**(A)** 2DoF inverted pendulum. **(B)** Trajectories of joints 1 and 2 in the process of regaining balance in the experiment. Each point is supplemented with a spline curve. The gray areas are where the joints move in phase, the white areas are where they move in anti-phase. Joints 1 and 2 move in anti-phase in the process of regaining balance, as in the simulation with tacit learning.

We conducted an experiment on a real 2DoF inverted pendulum with the same block diagram as in the simulation. The transfer matrix was calculated based on the physical parameters of the pendulum, whereas the gains in the block diagram were determined by trial and error. The experimental conditions can be seen in the Supplementary Video. Figure 7A shows the actual 2DoF inverted pendulum. Figure 7B shows the trajectories of joints 1 and 2 in the process of regaining balance. The pendulum becomes upright by moving joints 1 and 2 in anti-phase, as in the simulation with tacit learning in MRM-space.

### 3.5. Discussion of standing balance control

The pendulum can remain upright against larger disturbances as the coefficient of tacit learning in MRM-space is increased (see Figure 6). However, a problem is that the energy consumption per unit time in same disturbance also increases as the coefficient is increased. Another problem is that, although we did not analyze the stability of this system, too large an integral tacit learning coefficient makes the system unstable (see Shimoda et al., 2012). To regain balance efficiently after a disturbance, it is necessary to change the tacit learning coefficient according to the disturbance.

**Figure 8:**
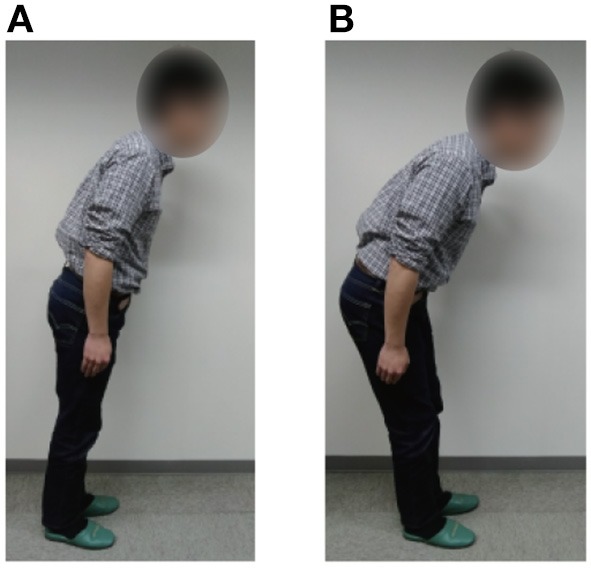
Standing balance control strategies of a person (Horak and Nashner, 1986; Runge et al., 1999; Robinovitch et al., 2002). **(A)** Ankle strategy: the person mainly moves the ankle joints and can balance against small disturbances only. **(B)** Hip strategy: the person moves the hip and ankle joints in anti-phase and can balance against larger disturbances. The participant of this figure gave informed consent to appear on the current work.

It is well-known that people change their standing balance strategies between ankle and hip strategies (Horak and Nashner, 1986; Runge et al., 1999; Robinovitch et al., 2002) according to the prevailing disturbances. Each strategy is shown in Figure 8. The ankle strategy is a standing balance control method in which the person mainly moves the ankle joints in response to a relatively small disturbance, whereas the hip strategy is a standing balance control method in which the person moves the hip and ankle joints in anti-phase in response to a larger disturbance. The movements involved in the hip strategy are similar to those of the 2DoF pendulum when tacit learning is applied in MRM-space. It is interesting that our method of adjusting signals in MRM-space with tacit learning has something in common with a human strategy.

## 4. Bipedal walking control on flat plane with 27DoF humanoid robot

In section 3, we discussed the use of standing balance control of allow a 2DoF pendulum to react to disturbances. In this section, we add a “top-down” signal to include intentional behavioral changes. We apply the same control strategies to a 27DoF humanoid robot walking control with the added top-down signal. The weight and the length of segments of the robot is decided based on the NASA biometric research (NASA). We show through simulation and experiment that the signal added to the controller in MRM-space plays the role of behavioral intentions to change walking direction while maintaining walking balance.

### 4.1. Bipedal walking control structure

**Figure 9:**
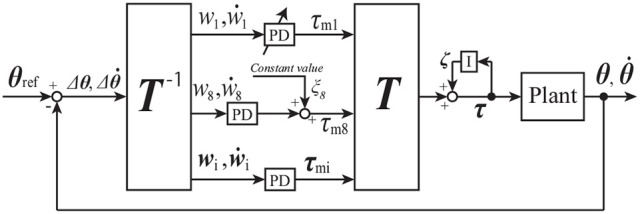
Block diagram for bipedal walking control of 27DoF robot; see section 4.1 for details.

Figure 9 shows a bipedal walking controller designed by using the transfer matrix **T** and tacit learning. Terms **θ**, $\overset{\cdot }{\theta }\in R^{27}$ are vectors of state variables. Terms $\theta_{ref}\in R^{27}$ is a vector of angle references. The torque vector **τ** ∈ **R**^27^ for each joint is represented as

(20) $$\begin{array}{lll} \tau & = & TAT^{-1}\Delta \theta +TBT^{-1}\Delta \overset{\cdot }{\theta }+Tc+\zeta , \end{array}$$

(21) $$\begin{array}{lll} \tau & = & {\left[\begin{array}{ccc} \tau_{1} & \cdots & \tau_{27} \end{array}\right]}^{T}, \end{array}$$

(22) $$\begin{array}{l} \begin{array}{c} \zeta =\\ diag\left(\left[\begin{array}{llll} 0\;\cdots & k_{lankle} & k_{rankle} & \cdots \;0 \end{array}\right]\right)\int \tau dt, \end{array} \end{array}$$

where **ζ** is a vector that consists of the integral values of tacit learning, and tacit learning is applied to only the left and right ankle joints. The balance in the sagittal plane is maintained by tacit leaning, as in Shimoda et al. (2013). Terms *k*_*lankle*_ and *k*_*rankle*_ are the integral coefficients for the left and right ankle joints, respectively. Terms Δ**θ** and $\Delta \overset{\cdot }{\theta }$ are

(23) $$\begin{array}{l} \Delta \theta =\left[\begin{array}{c} \theta_{ref}-\theta \end{array}\right],\;\Delta \overset{\cdot }{\theta }=-\overset{\cdot }{\theta }. \end{array}$$

Terms **A** and **B** are diagonal matrices:

(24) $$\begin{array}{l} \begin{array}{c} A=diag\left(\left[\begin{array}{cccc} k_{p1} & k_{p2} & \cdots & k_{p27} \end{array}\right]\right),\\ B=diag\left(\left[\begin{array}{cccc} k_{d1} & k_{d2} & \cdots & k_{d27} \end{array}\right]\right), \end{array} \end{array}$$

where *k*_*p*1_, …, *k*_*p*27_ are values obtained by multiplying the eigenvalues of each mode by 10, and *k*_*d*1_, …, *k*_*d*27_ are values obtained by multiplying the eigenvalues of each mode by 0.1. The eigenvalues are given by the mechanical resonance mode of the robot.

Term **c** is a vector that consists of the constant value of the torque of the eighth mode and is given by

(25) $$\begin{array}{l} c={\left[\begin{array}{ccc} 0\;\cdots & \xi_{8} & \cdots \;0 \end{array}\right]}^{T}, \end{array}$$

where ξ_8_ is a constant that can be adjusted manually.

The transfer matrix **T** ∈ **R**^27 × 27^ of the 27DoF robot can be calculated using the method given in section 2.1. The movement of all modes can be seen in the Supplementary Video. In controlling the robot behavior, we focus on two specific modes from all the modes, namely the first and eighth modes (see Figures 1 C,D). We expect to realize two specific types of walking. Adjusting only the signal of the first mode while changing the P gain *k*_*p*1_ can make the robot change its walking velocity on a flat plane. Adjusting the signal of the eighth mode with the constant value **c** can make the robot turn left and right on a flat plane with fixing *k*_*p*1_ for the robot to walk forward.

### 4.2. Bipedal walking simulation and results

**Figure 10:**
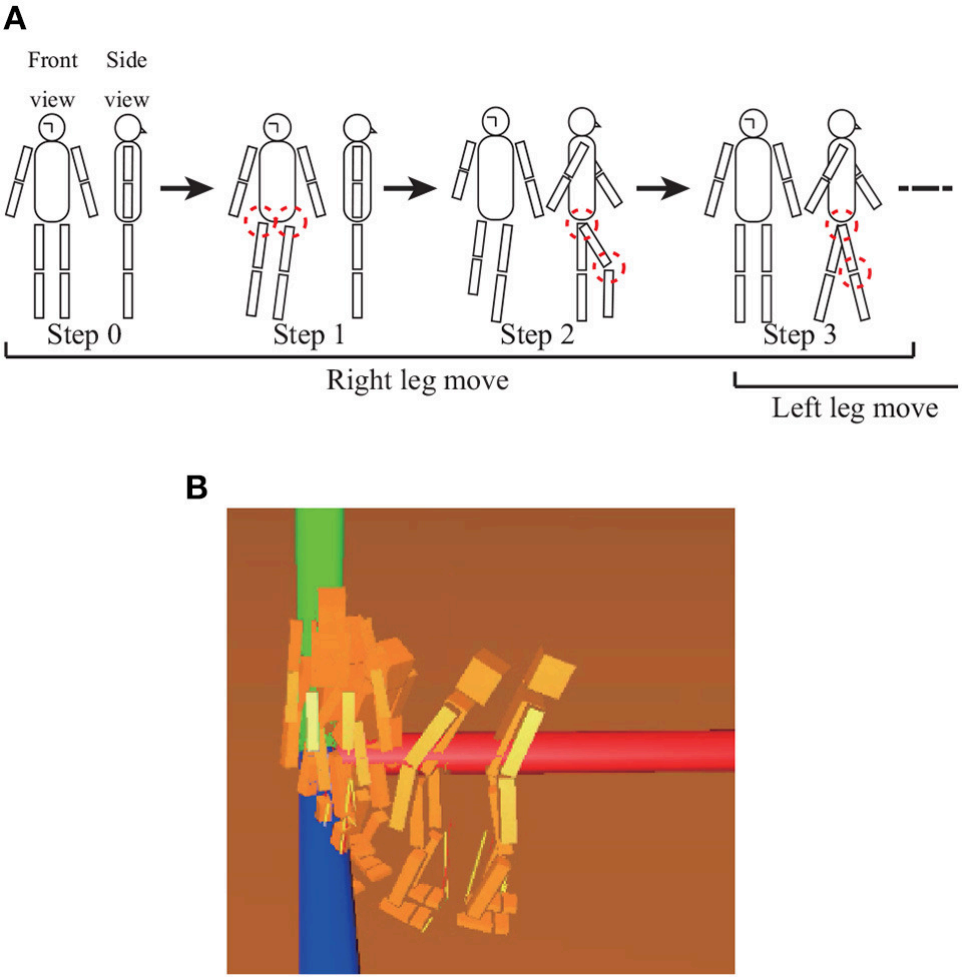
**(A)** Posture of each target set, for which the joints enclosed by dashed circles are controlled. Steps 5–7 (not shown) are the bifrontally symmetric postures of steps 1–3. Steps 4 and 8 (not shown) involve waiting for a time while holding the posture of the corresponding previous target set. References for joints are given in Table 2. **(B)** Overview of walking simulation for adjusting the eighth mode. By adding positive constant values to the eighth mode, the robot can turn left.

Bipedal walking is performed as shown in Figure 10A, with one cycle of walking consisting of eight steps. The integral coefficients for the left and right ankle joints are *k*_*lankle*_ = *k*_*rankle*_ = 1.0*e* − 4. The references for each joint at each step in the simulation are described in Table 2. After finishing one cycle, the reference returns to the beginning of the cycle.

**Table 2:** Target set for bipedal walking control simulation.

		**Target angle [rad]** × 10^**−1**^	
**Step**	**Description**	**Left leg**	**Right leg**
Step 0	Standing posture	–	–
		Hip	Hip
Step 1	Balance on left leg	(−0.1)	(0.1)
			Hip Knee
Step 2	Right leg up	–	(−9.0) (9.0)
			Hip Knee
Step 3	Right leg down	–	(−4.5) (4.5)
Step 4	Balance on both leg	–	–
		Hip	Hip
Step 5	Balance on right leg	(0.1)	(−0.1)
		Hip Knee
Step 6	Left leg up	(−9.0) (9.0)	–
		Hip Knee
Step 7	Left leg down	(−4.5) (4.5)	–
Step 8	Balance on both leg	–	–

Each step shifts to the next step under specific conditions. When the robot raises a foot, that step shifts to the next step when a knee joint angle of the robot equals the reference of the knee joint angle. When the robot puts a foot down, that step shifts to the next step when the sole of the foot touches the ground. If the robot falls down while walking, the robot is moved to the initial position while holding the integral values of tacit learning.

(i) Walking forward and backward

**Figure 11:**
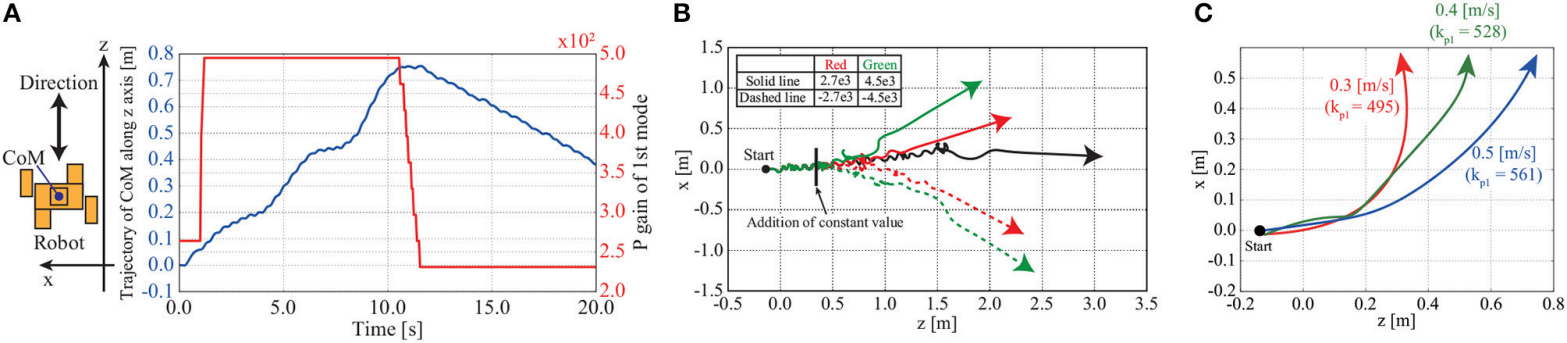
Trajectory and time series of CoM position in turning and walking forward and backward in simulation: **(A)** Time series of CoM position in the direction in which the robot walks and the P gain of the first mode in the simulation. In the early stage of walking, the P gain is set as *k*_*p*1_ = 270.0. The robot walks forward and backward according to changes in the P gain of the first mode. **(B)** Trajectories of robot CoM in walking simulation with adjusting the movement of the eighth mode. The robot walks from the left to the right of the figure. The black line is a trajectory of the robot walking forward without adjusting the movement of the eighth mode. The walking direction is changed depending on the constant value. The robot turns more as the constant value is increased. **(C)** Directions of robot CoM in turning with different walking velocities. Solid lines represent walking directions. The constant value is set as ξ_8_ = 4.5*e*3. Walking velocity can be changed in turning behavior with different P gain *k*_*p*1_, and the walking direction is affected by walking velocity.

Figure 11A shows time series of the CoM position in the direction in which the robot walks and the P gain *k*_*p*1_ used to adjust the movement of the first mode. In the early stage of walking, the P gain is set as *k*_*p*1_ = 270.0. It can be seen that the robot walks forward and backward according to the adjustment of the P gain. An overview of the simulation can be seen in the Supplementary Video.

(ii) Turning left and right

Figure 10B shows an overview of the walking simulation adjust the movement of the eighth mode by adding positive constant values to the signal of the eighth mode with an appropriate P gain *k*_*p*1_ to walk forward. The robot can be seen turning left.

Figure 11B shows the trajectories of the CoM of the robot from the top view; the robot walks from the left of the figure to the right with teh appropriate P gain *k*_*p*1_. From Figure 11B, it can be seen that the walking direction is changed depending on the constant value used to adjust the movement of the eighth mode. The robot turns more as the constant value is increased. An overview of the simulation can be seen in the Supplementary Video.

Figure 11C shows the trajectories of the CoM of the robot from the top view; the robot walks from the left of the figure to the right with a fixed constant value ξ_8_ = 4.5*e*3. From Figure 11C, it can be seen that P gain *k*_*p*1_ is a key factor that affects the velocity in turning behaviors, and the walking direction is changed.

### 4.3. Bipedal walking experiment and results

We conducted not only simulations but also walking experiments by using an actual robot, namely the humanoid robot NAO made by Aldebaran. This has 25DoF, and we can control the angle of each of its joints. NAO is controlled by angle inputs rather than torque inputs. We designed a control structure that generates reference joint angles by using the transfer matrix **T**.

The reference joint angle for a wide variety of walking can be described as

(26) $$\begin{array}{l} \phi^{\prime }=T^{†}\left[\left[\begin{array}{llll} k_{p1} & & & 0\\ & 1.0 & & \\ & & \ddots & \\ 0 & & & 1.0 \end{array}\right]T^{†}{}^{-1}\phi +\left[\begin{array}{c} 0.0\\ \vdots \\ \alpha_{8}\\ \vdots \\ 0.0 \end{array}\right]\right]\\ if\;k_{p1}=1.0\;and\;\alpha_{8}=0.0,\;\phi^{\prime }=\phi , \end{array}$$

where **ϕ** ∈ **R**^25^ is a vector that consists of joint-angle references for walking, and **ϕ**′ ∈ **R**^25^ is an adjusted vector. Term **T**^†^ is a transfer matrix modified from the transfer matrix **T** to fit the DoF of NAO. Term *k*_*p*1_ works like the P gain in the simulation of walking forward and backward. Term α_8_ works like the constant value in the simulation of turning. Walking balance is acquired by applying tacit learning to joint space in the same way as 2DoF inverted pendulum postural control.

Experiments are conducted using the same scheme as that shown in Figure 10A. The target joint angles at each step in the simulation are described in Table 3. Each step shifts to the next step under specific conditions. When NAO raises a foot, that step shifts to the next step when the knee joint angle of the robot equals the reference knee joint angle. When NAO puts a foot down, that step shifts to the next step when the sole of the foot touches the ground.

**Table 3:** Target set for bipedal walking control experiment.

		**Target angle [rad]**	
**Step**	**Description**	**Left leg**	**Right leg**
Step 0	Standing posture	–	–
		Hip	Hip
Step 1	Balance on left leg	(−0.1*e* − 1)	(0.1*e* − 1)
			Hip Knee
Step 2	Right leg up	–	(−0.8) (1.0)
			Hip Knee
Step 3	Right leg down	–	(−0.4) (0.7)
Step 4	Balance on both leg	–	–
		Hip	Hip
Step 5	Balance on right leg	(0.1*e* − 1)	(−0.1*e* − 1)
		Hip Knee
Step 6	Left leg up	(−0.8) (1.0)	–
		Hip Knee
Step 7	Left leg down	(−0.4) (0.7)	–
Step 8	Balance on both leg	–	–

(i) Walking forward and backward

**Figure 12:**
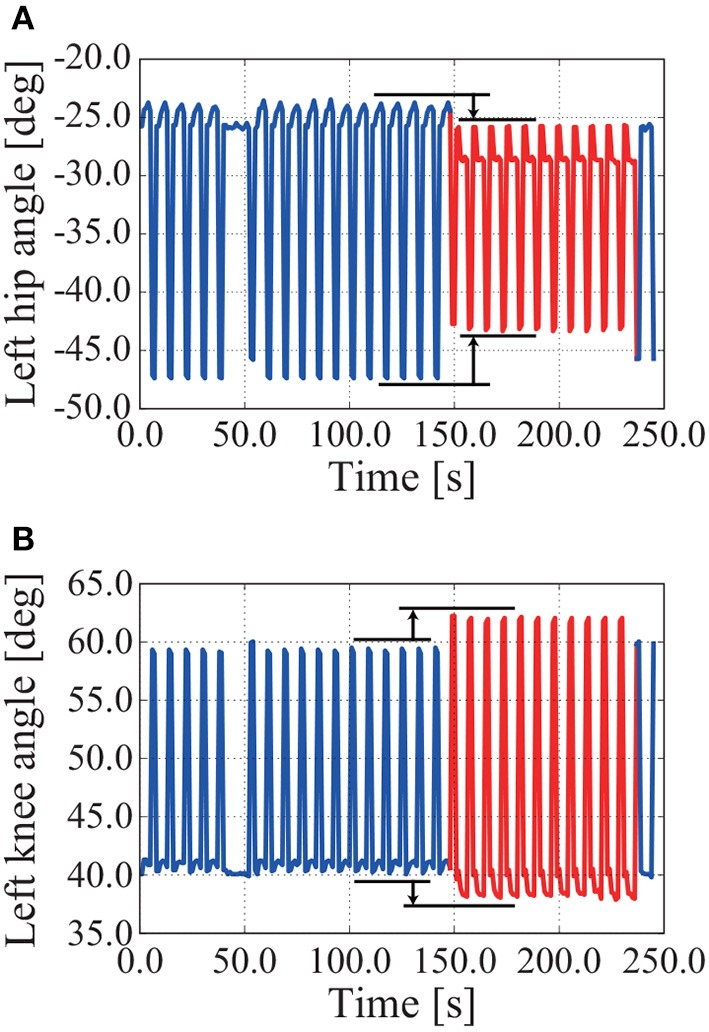
Time series of left hip and left knee joint angles in an experiment involving walking forward and backward. The P gain *k*_*p*1_ for adjusting the movement of the first mode changes as follows: *k*_*p*1_ = 1.0 for 0–150 s, *k*_*p*1_ = 1.1 for 150–240 s, and *k*_*p*1_ = 1.0 for 240–250 s. NAO walks forward for 0–150 s and backward for 150–240 s. When walking is switched from forward to backward by adjusting the movement of the first mode, the hip joint angle decreases and the knee joint angle increases. **(A)** Left hip angle. **(B)** Left knee angle.

NAO can walk forward and backward when we adjust only the movement of the first mode by adjusting its gain. An overview of the experiment can be seen in the Supplementary Video. Figure 12 shows time series of the left hip joint and left knee joint angles while walking forward and backward. When walking is switched from forward to backward by adjusting the movement of the first mode, the hip joint angle decreases and the knee joint angle increases (see around 150 s in Figure 12).

(ii) Turning right and left

**Figure 13:**
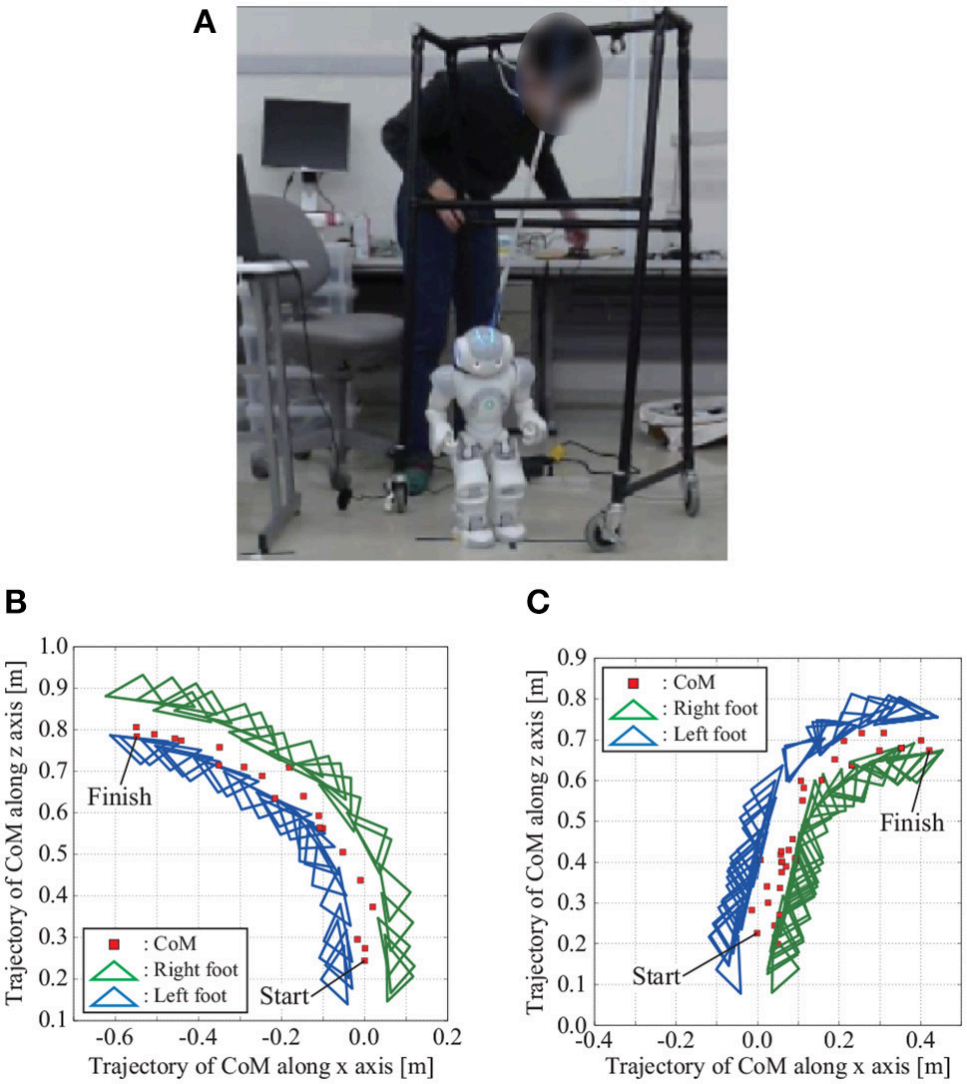
Overview of walking experiment with NAO, and CoM and foot trajectories when NAO turns left and right in the experiment. **(A)** Overview of walking experiment with NAO. **(B)** Turning left: NAO can turn left when the constant value α_8_ for adjusting the movement of the eighth mode is positive. **(C)** Turning right: NAO can turn right when the constant value α_8_ for adjusting the movement of the eighth mode is negative. The participant of this figure gave informed consent to appear on the current work.

**Figure 14:**
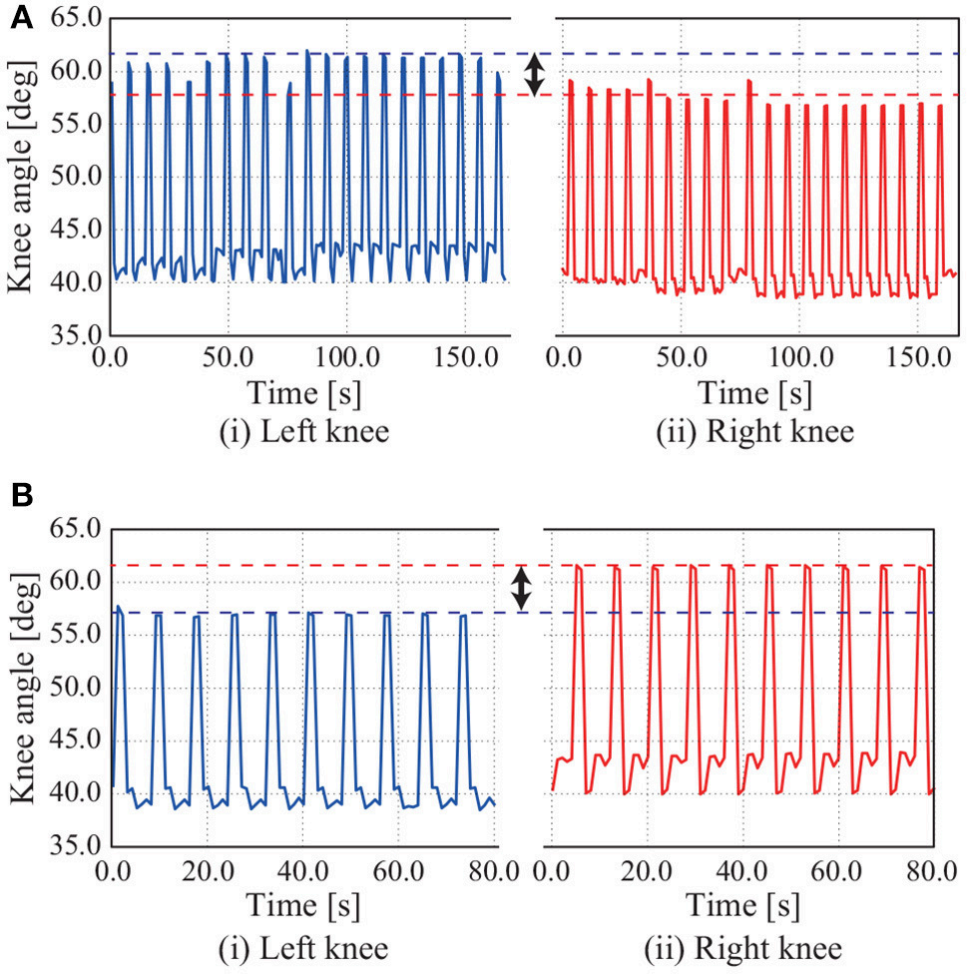
Time series of knee joint angle in turning experiment: **(A)** turning right; **(B)** turning left. When NAO turns right, the amplitude of the left knee joint angle is bigger than that of the right knee joint angle in adjusting the movement of the eighth mode by the constant value α_8_ = −25.0. When the constant value is negative, NAO turns left.

Figure 13A shows NAO turning left adjust the movement of the eighth mode by adding a positive constant value to the signal of the eighth mode with an appropriate P gain *k*_*p*1_ to walk forward. Figures 13B,C shows the trajectories of the CoM and feet when the movement of the eighth mode is adjusted with positive and negative constant values. Figure 14 shows time series of the knee angles when NAO turns left and right.

NAO can turn left and right by adjusting the movement of the eighth mode, as in the simulation. As shown in Figure 14A, the amplitude of the left knee joint angle is bigger than that of the right knee joint angle in adjusting the movement of the eighth mode by the constant value α_8_ = −25.0, whereupon NAO turns left. The converse holds in Figure 14B.

### 4.4. Discussion of bipedal walking control

The robot NAO could turn left and right by adjusting the movement of the eighth mode, that is, adjusting the bending of the robot and NAO at the waist on the frontal plane by adjusting the constant value. Adjusting the movement of the eighth mode deflected the CoM to the left-hand or right-hand side of the body, whereupon one foot took a larger step than did the other foot. This phenomenon can be confirmed from the fact that the amplitude of the left knee joint angle was bigger than that of the right knee joint angle (see Figure 14A).

The robot and NAO could walk forward and backward by adjusting the movement of the first mode. The robot and NAO differed in how the gain was adjusted, but we consider this to be because the gains of the other modes differed between the robot and NAO. When the movement of the first mode is adjusted, the degree to which the legs are opened is adjusted, whereupon the position at which the foot touches the ground can be adjusted. This can be confirmed from the fact that the hip joint angle decreases and the knee joint angle increases when walking switches from forward to backward (see Figure 12).

It is normally difficult to make a robot walk in a wide variety of ways because that necessitates designing a plurality of controllers and preparing references concerning each combination of individual joints. Instead, our method realizes turning and walking forward and backward smoothly by adjusting a few symbolized parameters in MRM-space without having to care about each combination of 27 joints and switching controllers. This is because there is a pattern of movements according to the mode, and the pattern to adjust is easy to understand visually.

A person can change behavior while caring intentionally only about “turn left and right” or “forward and backward.” Likewise, our method can adjust signals and realize behavior without caring about each joint, which is important in considering human-like movement in the robot under consideration.

## 5. Discussion

As discussed in section 1, the aim of this paper is to clarify the mechanisms of generating automatic motor commands to achieve a symbolized behavioral purpose. Our approach is to develop an artificial controller that embodies those mechanisms and derive the important features of that function to assess the extent to which the robot behavior is human-like.

We reasoned that the key problem in developing a controller with those mechanisms would be realizing bio-mimetic adaptation with two-way behavioral adaptation. The first way is selecting the appropriate behavior that progresses in a top-down manner, and the second is adjusting the behavior according to the environment, which is a bottom-up process that progresses through body–environment interactions.

In the preliminary study, we discussed the standing balance control of a 2DoF inverted pendulum to introduce our control strategies, focusing only on the bottom-up adaptation process. In our control strategy, the control signals to each joint are transferred to another space computed by using the mechanical resonance modes, whereby we can easily understand the behavioral pattern that each control signal creates. We applied tacit learning in MRM-space and developed the controller to maintain standing against various levels of disturbance. The simulation and experimental results showed that the standing balance control capability was increased when the 2DoF inverted pendulum was controlled using MRM-space, suggesting that the simple adaptation mechanism is enough to improve the behavioral performance of standing balance when behavior control is conducted in a space where we can set the direction of behavior by adjusting to the change of the disturbance. The similarities between the mechanical resonance modes used in this system and the hip and ankle motion strategies of people must be another indication of the importance of reacting to a wide range of disturbances in a space in which the body parameters are well represented.

In the bipedal walking study using NAO, we discussed two-way adaptation. To represent the top-down process in which the behavioral purpose is selected, we chose the appropriate resonance mode with which to adjust the behavior to the desired one. As described in Figure 13, when we adjust the parameter of the eighth mode, the robot begins to turn left and right while maintaining walking balance. Walking balance was maintained by tacit learning applying the method in Shimoda et al. (2013) to joint space in the same way as 2DoF inverted pendulum control as a bottom-up process. In the simulation and the experiments, we succeeded in changing the behavior to turn left/right and go forward/backward by stimulating different mechanical resonance modes.

We consider that the signals added to the specified mode control can be treated as symbolized behavioral purpose in our study. The detailed commands to the joint control were created in two processes, namely, transferring the control signal from MRM-space to joint space, and tacit learning for maintaining walking balance while reacting to environmental inputs. Therefore, these results suggest that one-dimensional signal change can create the complicated combinations of the 25DoF of NAO required to change the behavior when the appropriate mode is stimulated.

Joint control is much more complicated in people than it is in robots because complicated combinations of muscles are required in the former. The notion of muscle synergy introduced in section 1 shares the same features as those of the mechanical resonance mode used in our method for robot control because the muscle-synergy space represents the behavioral features of body mechanisms and contributes to adjusting behavior in our case by using lower-dimensional signals.

**Figure 15:**
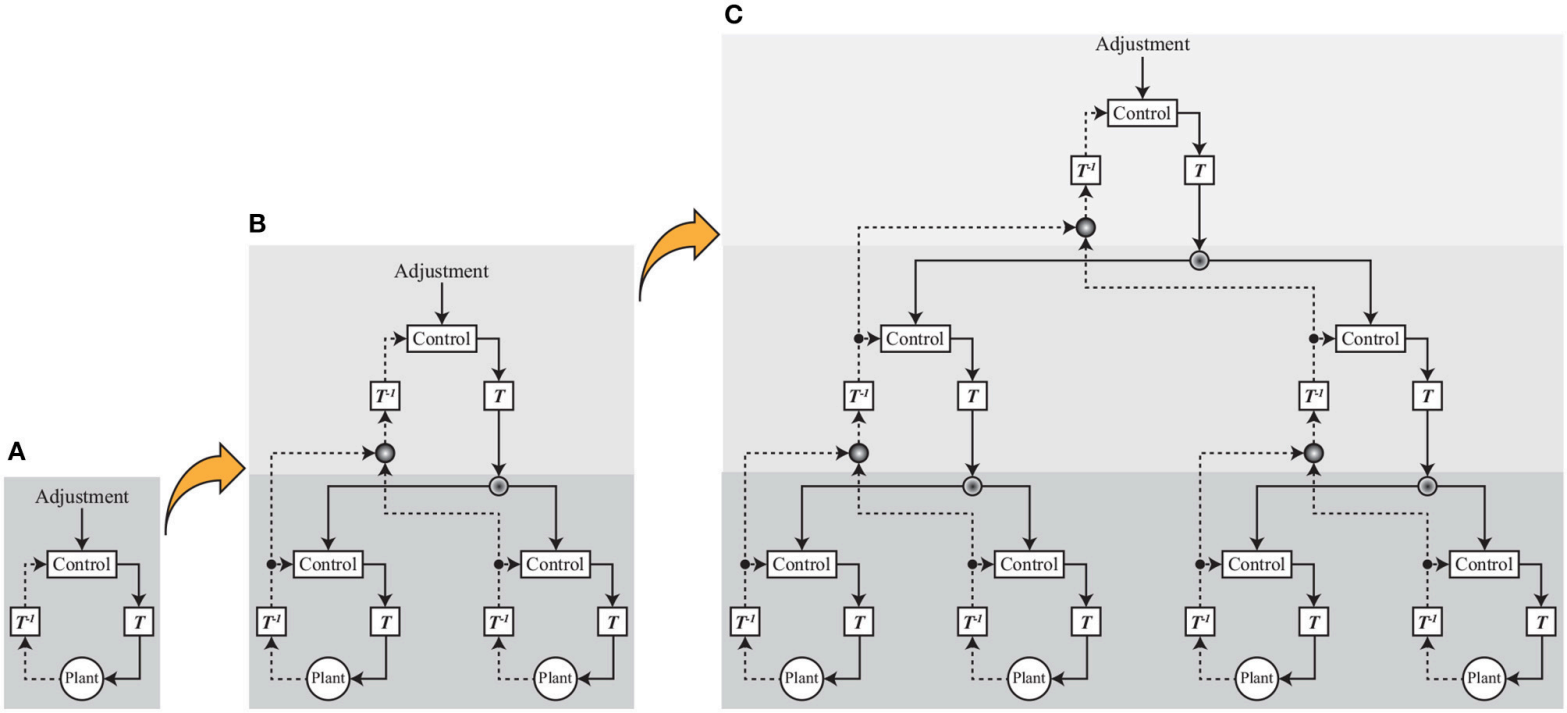
Neuro-synergy system concept. **(A)** One layer. This is a schematic diagram that is used in the simulations and experiments. **T**^−1^ plays the role of integrating complex signals from the environment and generating semantic information, and **T** plays the role of converting semantic information to specific control signals. In this paper, standing balance and walking control is realized by adjusting part of the signals in a transfer space. **(B)** Two layers. Part of the integrated signals is integrated, and an upper layer is formed. Control and adjustment are conducted in the upper layer, and the output signals from the upper layer play the role of adjusting in the lower layer. **(C)** Three layers. The layer step-up is accumulated to form a larger network, and we can produce various behaviors from the more-symbolized behavioral target.

These results and discussion suggest that transferring the signals to the appropriate control space is the key process for reducing the complexity of the signals from the environment. Discussing muscle synergy in a human controller and the mechanical resonance mode is only one step to the final output. If such steps were to be accumulated to form a larger network as described in Figure 15, we could form various behaviors from the more-symbolized behavioral target such as “go to the station.”

We reason that the extent to which a symbolized target that is represented by the lower-dimensional signals is used to create the robot behavior is the critical assessment for evaluating the extent to which the robot behavior is human-like. The results in this paper are just one step up from pure motor-control signals, implying far from human-like behavior. Further discussion is required to elevate the proposed system to using more-symbolized behavioral targets such as “go to the station.” A key problem is automatic creation of the mechanical resonance mode. Non-linear transfer for more complicated environment is another important problem. Even in the control of human behavior, the process of creating muscle synergy remains mysterious. We are now on the way to clarifying the process to a morehierarchical system in both physiological and artificial ways.

## Author contributions

MT explained the concept of mechanical resonance mode of the robot to SO. MT made a model of robot control structure using the mechanical resonance mode. MH and SS explained the concept of a biological learning method called tacit learning and gave advices on how to use tacit learning to SO. To clarify the mechanisms that generate automatic motor commands to achieve symbolized behavior, SO made the concept of adaptive control method using mechanical resonance mode and a biological learning method called tacit learning, designed a control structure to realize symbolized behavior purpose in discussions with FA, MH, YH, MT, and SS. SO conducted standing balance control simulations and bipedal walking control simulations with a 2DoF inverted pendulum and 27DoF humanoid robot, respectively. In discussions with FA, MH, YH, and SS, SO conducted standing balance control experiments and bipedal walking control experiments with an actual 2DoF inverted pendulum and an actual humanoid robot, respectively. SS helped in using the actual pendulum and humanoid robot in experiments.

### Conflict of interest statement

The authors declare that the research was conducted in the absence of any commercial or financial relationships that could be construed as a potential conflict of interest.

## Footnotes

^1^Roomba. *iRobot Corporation*. Available online at: https://www.irobot.com/

^2^Atlas. *Boston Dynamics*. Available online at: https://www.bostondynamics.com/

^3^Amazon Echo. *Amazon.com, Inc*. Available online at: https://www.amazon.com/dp/B00X4WHP5E

^4^Google Home. *Google LLC*. Available online at: https://store.google.com/product/google_home

^5^NAO. *SoftBank Robotics*. Available online at: https://www.ald.softbankrobotics.com/en/robots/nao
